# The Yeast Tor Signaling Pathway Is Involved in G2/M Transition via Polo-Kinase

**DOI:** 10.1371/journal.pone.0002223

**Published:** 2008-05-21

**Authors:** Akio Nakashima, Yoshiko Maruki, Yuko Imamura, Chika Kondo, Tomoko Kawamata, Ippei Kawanishi, Hideki Takata, Akira Matsuura, Kyung S. Lee, Ushio Kikkawa, Yoshinori Ohsumi, Kazuyoshi Yonezawa, Yoshiaki Kamada

**Affiliations:** 1 Biosignal Research Center, Kobe University, Kobe, Japan; 2 CREST, Japan Science and Technology Agency, Kawaguchi, Japan; 3 Division of Molecular Cell Biology, National Institute for Basic Biology, Okazaki, Japan; 4 Department of Geriatric Research, National Institute for Longevity Sciences, Obu Aichi, Japan; 5 Laboratory of Metabolism, Center for Cancer Research, National Cancer Institute, National Institutes of Health, Bethesda, Maryland, United States of America; University of Giessen Lung Center, Germany

## Abstract

The target of rapamycin (Tor) protein plays central roles in cell growth. Rapamycin inhibits cell growth and promotes cell cycle arrest at G1 (G0). However, little is known about whether Tor is involved in other stages of the cell division cycle. Here we report that the rapamycin-sensitive Tor complex 1 (TORC1) is involved in G2/M transition in *S. cerevisiae*. Strains carrying a temperature-sensitive allele of *KOG1* (*kog1-105*) encoding an essential component of TORC1, as well as yeast cell treated with rapamycin show mitotic delay with prolonged G2. Overexpression of Cdc5, the yeast polo-like kinase, rescues the growth defect of *kog1-105*, and in turn, Cdc5 activity is attenuated in *kog1-105* cells. The TORC1-Type2A phosphatase pathway mediates nucleocytoplasmic transport of Cdc5, which is prerequisite for its proper localization and function. The C-terminal polo-box domain of Cdc5 has an inhibitory role in nuclear translocation. Taken together, our results indicate a novel function of Tor in the regulation of cell cycle and proliferation.

## Introduction

Cell growth (increase in cell mass) and proliferation (increase in cell number) are tightly linked with the cell's perception of its nutritional environment. Nutritional information is perceived by Target of rapamycin (Tor), a phosphatidylinositol kinase-related Ser/Thr kinase that plays a central role in controlling cell growth[1]. Tor signaling participates in two complexes; Tor complex 1 (TORC1) is sensitive to inhibition by rapamycin, whereas Tor complex 2 (TORC2) is not[2]. Yeast TORC1 consists of either of the two yeast Tor homologs, Tor1 or Tor2 together with co-factors Kog1, Lst8, and Tco89. With the exception of Tco89, these proteins are essential and widely conserved in eukaryotes. Rapamycin-sensitive Tor signaling mediated by TORC1 mainly regulates temporal aspects of cell growth: protein synthesis, amino acid uptake, autophagy induction. On the other hand, TORC2, consisting of Tor2, Avo1-3 and Lst8, regulates spatial aspects of cell growth, e.g. organization of actin cytoskeleton. TORC1 function is critical for protein synthesis activities such as translation initiation and ribosome biogenesis[3]–[6]. For example, mammalian TORC1 directly phosphorylates translation initiation factor (eIF) 4E binding protein 1 (4E-BP1) to abolish its binding and inhibitory activity toward eIF-4E[7]. Yeast TORC1 stimulates transcription of ribosomal RNA[4], expression of ribosomal protein genes via protein kinase A (PKA)[5], and maturation of the 60S ribosome[6]. It is also known that TORC1 is required for G1 progression, as rapamycin causes cell cycle arrest at early G1 (G0) stage[3]. G0 arrest caused by dysfunction of Tor is mainly explained by impairment of protein synthesis, because mutants of genes involved in early G1 progression (e.g. *cdc25*, *cdc33*, and *cdc35/cyr1*) display a similar phenotype, the stringent response of macromolecular synthesis[8], [9].

Type 2A phosphatase (PP2A) and PP2A-like phosphatase (Sit4) are identified as major downstream targets of the TORC1 pathway[1], [10]. These phosphatases form a complex with Tap42 under the control of TORC1[11]. Though Tap42 used to be believed to be a negative regulator of phosphatases, several lines of evidence recently suggest that Tap42-PP2A plays a positive role in phosphatase activity[10], [12]. Phosphotyrosyl phosphatase activator encoded by *RRD1* and *RRD2* also binds to Tap42-phosphatase complex in a Tor-dependent manner, suggesting that TORC1 may regulate substrate specificity of the phosphatases[13]. Involvement of PP2A in TORC1 signaling is also proposed in mammalian cells[14].

Recently, rapamycin has been studied as an anti-cancer drug, because it slows the proliferation of many cancer cell lines[1]. Therefore, it is an urgent issue to resolve how rapamycin-sensitive TORC1 activity influences cell proliferation. However, a detailed analysis of how Tor regulates the cell cycle through the control of cell growth has not yet been performed.

In this study we generated temperature-sensitive *KOG1* mutants to investigate the relationship of rapamycin-sensitive TOR signaling pathway to cell cycle progression. Genetic and biochemical analyses of *kog1* mutants led us the unexpected finding that TORC1 is involved in mitotic entry via regulation of polo-like kinase, Cdc5. Microscopy revealed that TORC1-PP2A pathway regulates the nuclear translocation of Cdc5, which is an indispensable step for its subsequent localization and function. Using these results, we present a model of how TORC1 facilitates nuclear transport of Cdc5 to promote mitotic entry.

## Results

**Figure 1:**
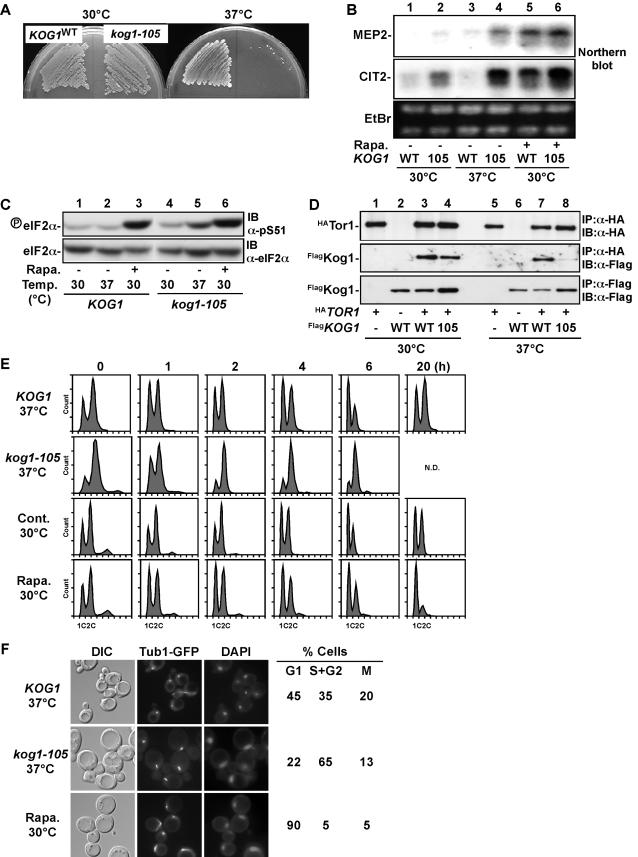
Characterization of *kog1-105*, a temperature-sensitive mutant of *KOG1* gene. (A) Wild-type (*KOG1*, YYK409) and *kog1-105* (YYK410) were grown on YEPD at 30°C or 37°C for 2 days. (B) Enhanced expression of starvation-induced gene in *kog1-105*. Cells grown at 30°C were incubated at 37°C or 30°C in the presence of 200 ng/ml of rapamycin (Rapa.) for 2 h. Total RNA was extracted and analyzed for expression of *MEP2* (top panel) and *CIT2* (middle panel) genes by northern blot. RNA amount was monitored by ethidium bromide staining (bottom panel). (C) Phosphorylation of eIF-2α is enhanced in *kog1-105*. Cells grown at 30°C were incubated as described in (b). Cell lysate was analyzed by immunoblot using an anti-phospho eIF-2α antibody (pS51, top panel) and an anti-eIF-2α peptide antibody (bottom panel). (D) Tor1-Kog1 association is unstable in *kog1-105*. HA-tagged Tor1 (^HA^Tor1) was immunoprecipitated from wild-type (YAN86) and *kog1-105* cells (YAN103) (top panel), and co-precipitated Flag-tagged Kog1 (^Flag^Kog1) was detected by immunoblot (middle panel). Noted that protein amount of ^Flag^Kog1 was similar in wild-type and *kog1-105* (bottom panel). (E) Wild-type (*KOG1* (YYK409)) and *kog1-105* (YYK410) cells grown on YEPD at 30°C were incubated at 37°C or 30°C in the presence of 200 ng/ml of rapamycin for the indicated times. DNA content was determined by FACS analysis. N.D.; not determined because of loss of viability. (F) Wild-type (*KOG1* (KLY4206)) and *kog1-105* (YYK834) cells expressing GFP-tagged tubulin (Tub1-GFP) were treated as in (e) for 4 h. Spindle formation and DNA (DAPI staining) were observed by immunofluorescent microscope. Percentage of G1 (unbudded cell), S+G2 (budded cell with short spindle), and M phase (large budded cell with long spindle) cells was also determined.

To closely study the rapamycin-sensitive TORC1 function, we generated temperature-sensitive mutants of *KOG1*, an essential component of TORC1[2], [15]. We obtained two temperature-sensitive alleles, *kog1-105* and *kog1-117* (Figure 1A). Since these two mutant alleles showed basically the same phenotype (data not shown), we chose *kog1-105* mutant for further study. At the non-permissive temperature, *kog1-105* cells showed similar phenotypes to rapamycin-treated cells. For example, *MEP2* (ammonium permease)[16] and *CIT2* (peroxisomal citrate synthase) genes[17] were highly expressed in *kog1-105* cell at 37°C (Figure 1B). Phosphorylation of translation initiation factor eIF-2α[18], [19] was also enhanced (Figure 1C). Consistent with these phenotypes, *kog1-105* exhibited impaired Tor1-Kog1 interaction at the non-permissive temperature, despite normal levels of Kog1 protein in the mutant strain (Figure 1D). These results suggest that incubation of *kog1-105* cell at non-permissive temperature causes dysfunction of TORC1.

**Figure 2:**
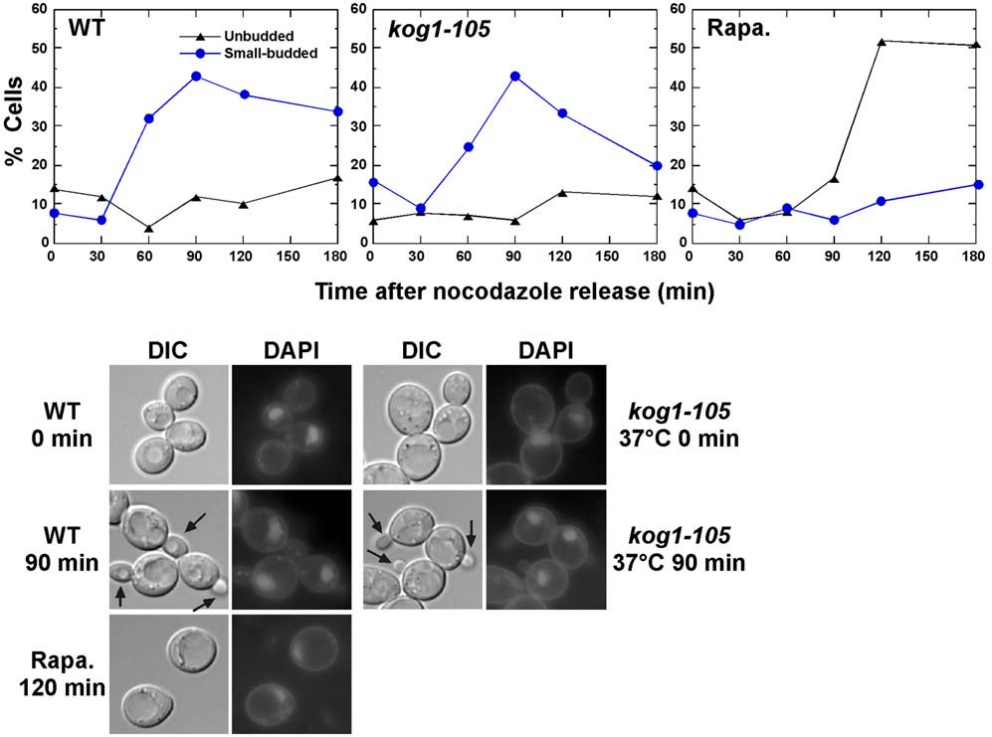
kog1-105 cell does not arrested at G1 at non-permissive temperature. Cell cultures (WT (YYK409) and *kog1-105* (YYK410)) blocked in metaphase with 15 µg/ml nocodazole were released into YEPD at 30°C (WT cont.), at 37°C (*kog1-105*), or 30°C in the presence of 200 ng/ml rapamycin (WT, rapa.). Budding indices (%) were determined to monitor the cell cycle progression. Cell image (DIC and DAPI staining) of the culture was shown. Arrow indicates small bud.

When treated with rapamycin, yeast cells terminally arrested at G1 with 1C DNA content (Figure 1E). It took longer than 6 h to achieve arrest at G1 by rapamycin, whereas it took only 1–2 h for reach G1 arrest by α-mating pheromone (data not shown), suggesting that cell cycle progression is delayed in rapamycin-treated cell. To our surprise, *kog1-105* cells did not arrest at G1 at 37°C; instead they stopped or slowed down cell cycle progression with 2C DNA content (Figure 1E). Characteristic to G2 phase these cells had medium to large buds with short spindles and undivided nuclei (Figure 1F). This unexpected result raised two questions: why do *kog1-105* cells not arrest at G1; and does rapamycin treatment or nutrient depletion also cause retardation at G2? To address the first question, yeast cell culture arrested with nocodazole at metaphase was released into fresh medium. Cells released into rapamycin-containing medium re-arrested at G1 as unbudded cells right after they exited mitosis (Figure 2). However, *kog1-105* cells released at non-permissive temperature progressed through G1, as judged by the emergence of small buds (Figure 2), indicating that *kog1-105* does not arrest at G1 at the non-permissive temperature. This result also implies that cell cycle stage delayed by *kog1-105* is one that occurs prior to the stage of arrest caused by nocodazole (metaphase). Autophagy, which is induced in nutrient-starved cells, was not induced in *kog1-105* at 37°C (data not shown), suggesting that incubation of *kog1-105* at the non-permissive temperature may not completely mimic nutrient starvation, which promotes G1 arrest.

**Figure 3:**
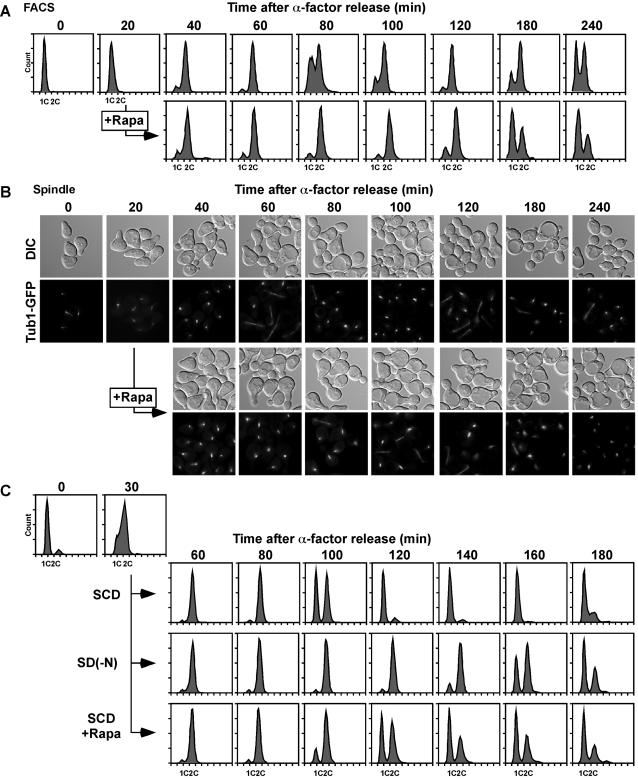
Rapamycin treatment and nitrogen-depletion induce prolonged G2 phase. (A) Cell culture (KLY4206) arrested at G1 by α-factor was released into YEPD. Rapamycin (200 ng/ml) was added at 20 min after release. DNA content was measured by FACS analysis. (B) Spindle formation was monitored by observation of GFP-tagged tubulin (Tub1-GFP). (C) Cell culture (W303-1B) arrested at G1 were released into SCD medium. Synchronous culture was collected at 30 min and re-released into SCD with/without Rapamycin (200 ng/ml) or nitrogen depleted medium (SD(-N)). Mating factor was added at 60 min for re-arrest at G1. DNA content was measured by FACS analysis.

To answer the second question, yeast cells released from α-factor block in G1 were treated with rapamycin. When released into rapamycin-containing medium, DNA replication was largely affected suggesting that most of the cells remained at G1 (data not shown). We thus added rapamycin 20 min after release. Control cell culture passed G2 at 40 min and entered mitosis at 60 min, as judged by FACS analysis, spindle formation, and bud indices (Figure 3A and B, Figure S1A). On the other hand, rapamycin-treated cells stayed at G2 at 80 min, and entered mitosis only at 100–120 min. The same experiments were performed using rapamycin-insensitive mutant, *TOR1-1* and *kog1-105*. Although wild-type cell exhibited G2 delay (80–100 min) by rapamycin as described, *TOR1-1* showed normal cell cycle progression even in the presence of rapamycin (Figure S1B). As shown in Figure 1E, *kog1-105* also showed retardation at G2 at the non-permissive temperature (90–150 min) (Figure S1C). Nutrient depletion also causes mitotic delay. When G1-arrested cells were released into the nitrogen-depleted medium, only small portion of cells entered S phase, confirming that nutrient is indispensable for G1/S transition (data not shown). Thus synchronously growing cells at S phase (30 min after release into SCD medium) were shifted into nitrogen-free medium (SD(-N)), and as a result a significant mitotic delay was observed (100–160 min time points) (Figure 3C). These results indicate that rapamycin-sensitive TORC1 function in response to nutrient signal is required for progression from G2 to mitosis.

**Figure 4:**
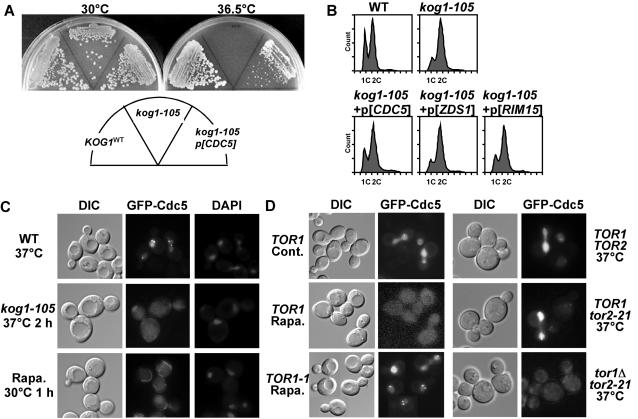
TORC1 regulates subcellular localization of Cdc5. (A) Overexpression of *CDC5* rescues growth defect of *kog1-105* cells. Wild-type (*KOG1* (YYK409)), *kog1-105* (YYK410), and *kog1-105* strains overexpressing *CDC5* (*p[CDC5]*) were grown on YEPD plates at 30°C or 36.5°C for 2 days. (B) Overexpression of *CDC5* suppresses G2-delay of *kog1-105* cells. Wild-type (YYK409), *kog1-105* (YYK410), and *kog1-105* cells harboring high-copy plasmid of the indicated genes were arrested at G1 and released into YEPD at 37°C. DNA content was measured by FACS analysis. FACS results of 120 min time point were shown. (C) Cells (WT (YYK409) and *kog1-105* (YYK410)) expressing EGFP-Cdc5 grown in SCD at 30°C were incubated at 37°C or 30°C with 200 ng/ml rapamycin for the indicated times. EGFP-Cdc5 was observed by fluorescent microscope. DNA (nucleus marker) was visualized by DAPI staining. (D) (Left panels) Wild-type (*TOR1* (JK9-3da)) and rapamycin-resistant *TOR1-1* (JH11-1c) cells expressing EGFP-Cdc5 grown in SCD at 30°C were treated with 200 ng/ml of rapamycin for 1 h. (Right panels) Wild-type (*TOR1TOR2* (JK9-3da)), *TOR1tor2-21* (SH121), and *tor1*Δ*tor2-21* (SH221) cells expressing EGFP-Cdc5 were incubated at 37°C for 2 h, before observation.

Next we screened for multicopy plasmids that rescued the growth defect of *kog1-105* at the non-permissive temperature. As a result we identified 3 multicopy suppressors, *ZDS1*, *RIM15*, and *CDC5* (Figure 4A, data not shown). Of these, only *CDC5*, the budding yeast polo-like kinase (Plk)[20], significantly relieved G2-delay of *kog1-105* cell (Figure 4B) when overexpressed (by its own promoter with a high copy plasmid). We further investigated whether Cdc5 acts downstream of TORC1.

Cdc5 localizes to and functions at the spindle-pole body (SPB), the bud neck and the nucleus[21]: SPB-localized Cdc5 plays multiple roles in mitosis[22], [23]; at the bud neck, Cdc5 has two roles—phosphorylating Swe1 at G2/M[24], and promoting cytokinesis at mitotic exit[25]; in the nucleus Cdc5 is involved in transcriptional regulation of *CLB2* gene[26]. Cdc5 localization at these sites was severely impaired in *kog1-105* cells at 37°C (Figure 4C). This was also disrupted when wild-type cells were treated with rapamycin, though the effect of rapamycin was canceled in *TOR1-1* cells (Figure 4D). Dysfunction of TORC2 in *TOR1tor2-21* ^ts^ strain did not exhibit mislocalization of Cdc5. These results suggest that TORC1 function (but not TORC2 function) is required for the subcellular localization of the yeast polo-kinase.

**Figure 5:**
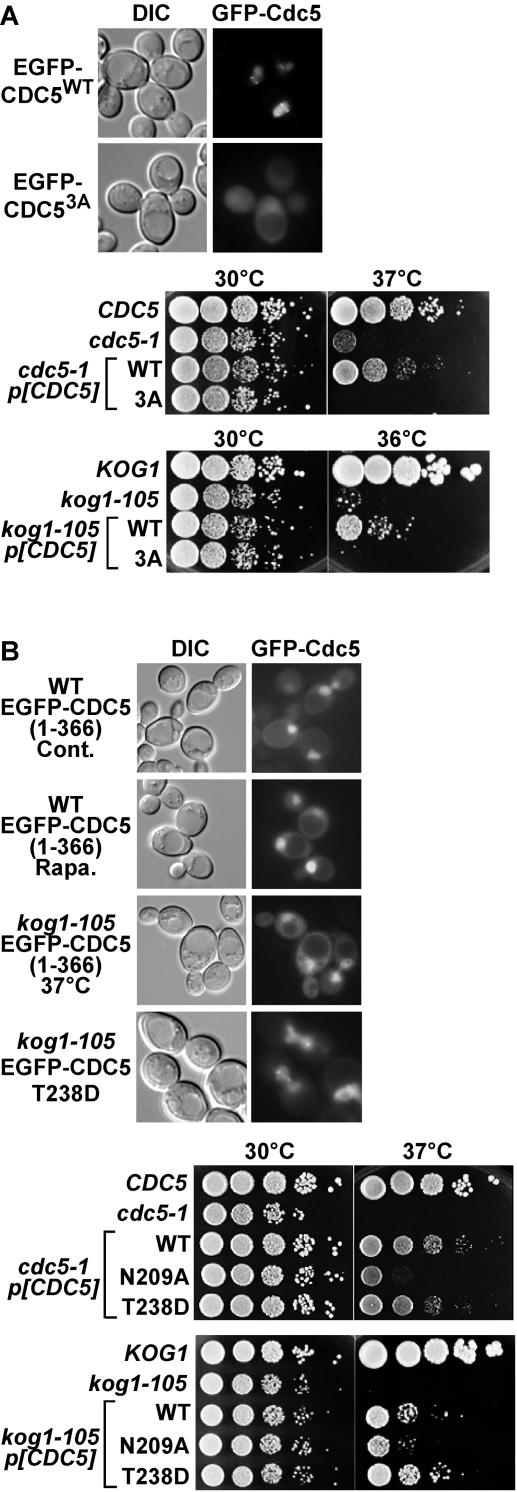
The C-terminus of Cdc5 has an inhibitory role in its nuclear translocation. (A) (Top) Cells (W303-1B) expressing EGFP-Cdc5 (wild-type or NLS mutant (3A)) grown in SCD at 30°C were observed by fluorescent microscope. (Bottom) Wild-type (*CDC5*, KLY1548), *cdc5-1* (KLY2156), and *cdc5-1* cells harboring the indicated high-copy EGFP-CDC5 plasmids were spotted onto YEPD plates and incubated at 30 or 37°C for 2 days. Wild-type (YYK409), *kog1-105* (YYK410), and *kog1-105* cells harboring the indicated EGFP-CDC5 plasmids were spotted onto YEPD. (B) (Top) Cells (*KOG1 and kog1-105*) expressing EGFP-tagged N-terminus of Cdc5 (Cdc5(1-366)) or T238D mutant (full length) grown in SCD at 30°C were treated with 200 ng/ml rapamycin or incubated at 37°C for 2 h. (Bottom) Experiment shown in the bottom panel of Figure 5A was carried out using the indicated high-copy EGFP-CDC5 plasmids.

Plks have a conserved nuclear localization signal (NLS) which mediates their nuclear translocation[27]. We confirmed that nucleocytoplasmic translocation of Cdc5 is mediated by classical nuclear import machinery, because nuclear import of Cdc5 was completely lost in mutants of karyopherin (Kap) complex (*srp1* and *kap95* for Kapα and Kapβ mutants, respectively)[28], and of the nuclear pore complex (*nup1*)[29] (Figure S2A). A mutant version of Cdc5p with a defective NLS (Cdc5^K171A/R172A/R173A^, designated as Cdc5^3A^) (Figure S2B) also showed abnormal localization (Figure 5A). Interestingly, mutation of the Cdc5 NLS (and disruption of nuclear import machinery) also altered localization of Cdc5 to the SPB and the bud neck, suggesting that nuclear translocation of Cdc5 is indispensable for its function, especially prerequisite for its proper localization to the SPB and the bud neck. This suggestion is supported by the fact that expression of Cdc5^3A^ was unable to rescue the growth defect of *cdc5-1* and *kog1-105* mutants (Figure 5A), though it was normally expressed (Figure S2B).

It is also known that the C-terminal polo-box domain (PBD) plays important roles in regulation of Plk[20]. We examined the effect of deletion of the C-terminus on TORC1-mediated localization of Cdc5. The N-terminal domain of Cdc5 (Cdc5(1-366)), which includes the whole kinase region and the NLS but lacks the PBD, localized to the nucleus (Figure 5B). However, Cdc5(1-366) could not localize to the SPB or the bud neck, confirming that the PBD is essential for Cdc5 localization to these sites[30]. Interestingly, Cdc5(1-366) was efficiently transported into the nucleus in rapamycin-treated cells and in the *kog1-105* mutant at 37°C (Figure 5B). The intra-molecular interaction of mammalian Plk1 between the N-terminal catalytic domain and the C-terminal PBD is disrupted by T210D substitution (corresponding to T238 of the yeast Cdc5)[31], [32]. We tested the effect of this mutation on Cdc5 localization in yeast, and found that GFP-Cdc5^T238D^ localized normally in *kog1-105* cells (Figure 5B). Cdc5^T238D^ can function normally, because it complemented *cdc5-1* cells and rescued growth defect of *kog1-105* cells (Figure 5B). These results suggest that in the case of Tor inactivation the C-terminal domain (presumably the PBD) has an inhibitory role in nuclear translocation of Cdc5.

**Figure 6:**
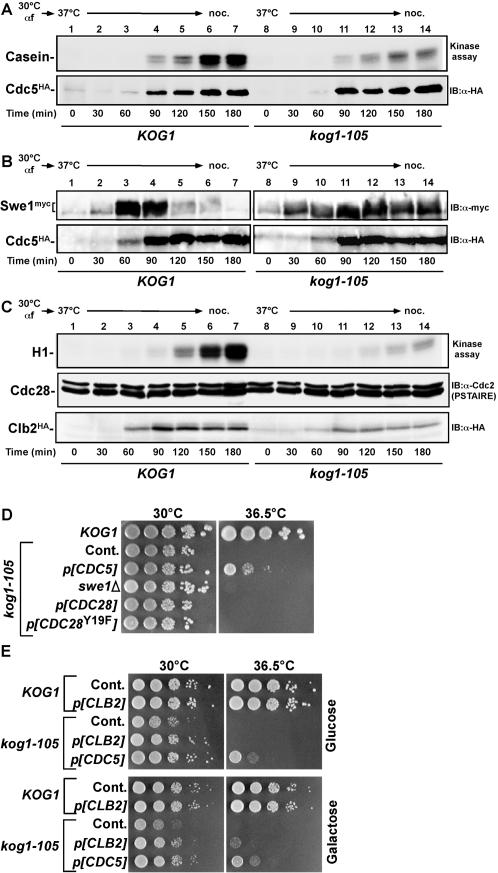
TORC1 mediates Cdc5 activity. (A) G1-arrested cells (*KOG1* (YYK540) and *kog1-105* (YYK541)), were released into nocodazole-containing YEPD medium at 37°C. Samples were harvested at the indicated times. Cdc5^HA^ protein was immunoprecipitated, and *in vitro* Cdc5 kinase assay was done using casein as a substrate. Note that induction of Cdc5 was normal in *kog1-105* cells (bottom panel). (B) G1-arrested cells (*KOG1* (YYK540) and *kog1-105* (YYK541)), were released into nocodazole-containing YEPD medium at 37°C. Swe1^myc^ [51] and Cdc5^HA^ proteins were detected by immunoblot. (C) G1-arrested cells (*KOG1* (YYK861) and *kog1-105* (YYK862)), were released as in (a). Cyclin-dependent kinase assay was performed using histone H1 as a substrate (top). Amount of Cdc28 and Clb2^HA^ was also shown (middle and bottom). (D) Wild-type (YYK409), *kog1-105* (YYK410), *kog1-105 swe1*Δ (YYK513), and *kog1-105* cells harboring the indicated plasmids (expressed under their own promoter) were spotted onto YEPD and incubated at the indicated temperature for 2 days. (E) Wild-type (YYK409), *kog1-105* (YYK410) cells harboring the high copy *CDC5* (p[*CDC5*], expressing under its own promoter) or *CLB2* (p[*CLB2*], expressing under GAL1 promoter) plasmids were spotted onto YEPD or YEPgalactose (overexpressing *CLB2*) and incubated at the indicated temperature for 2 days.

Protein kinase activity is indispensable for Cdc5 function. It is also required for suppression of *kog1-105* cells, because overexpression of *CDC5* ^N209A^, a kinase deficient allele, could not rescue growth defect of *kog1-105* cells (Figure 5B). We closely monitored Cdc5 activity as cells progressed from G1 to M. In wild-type cells, Cdc5 became robustly active at the late stages of the cell cycle (150-180 min time points)[33] (Figure 6A). In *kog1-105* cells, however, Cdc5 only exhibited a basal level of activity, though Cdc5 in *kog1* mutant accumulated as normal as in both wild-type. Cdc5 plays a critical role in hyper-phosphorylating and degrading Swe1 prior to G2/M transition[21], [22]. Swe1 negatively regulates mitotic Cdc28 (the yeast cyclin-dependent kinase) by inhibitory phosphorylation at tyrosine 19[21], [22]. During the normal cell cycle, Swe1 is induced in S phase, becomes progressively phosphorylated as cells proceed through the cell cycle, and is largely degraded prior to mitotic entry[21], [22]. The level of Swe1 remained stable in a *kog1-105* strain at non-permissive temperature (Figure 6B), and as a result Cdc28 activity was severely attenuated (Figure 6C). These results suggest that TORC1 regulates Cdc5 activity (and ensuing downregulation of Swe1 and activation of Cdc28) by controlling its subcellular localization.

In the budding yeast, the organization of the bud neck plays an important role in G2/M transition. Defects in bud and bud neck formation or perturbation of actin organization leads to a Swe1-dependent G2 delay called the morphogenesis checkpoint[34]. Since degradation of Swe1 protein was impaired in *kog1-105* mutant, we examined whether morphogenesis checkpoint is involved in phenotype of *kog1* cells. Deletion of *SWE1* or expression of *CDC28* ^Y19F^ (a mutant of inhibitory phosphorylation site by Swe1) in a *kog1-105* background did not rescue cell growth at the restrictive temperature (36.5°C) (Figure 6D). At semi-restrictive temperature (34°C) *SWE1* deletion could weakly rescue *kog1* cells (Figure S3C). When monitored Hsl7-GFP as a morphogenesis-checkpoint-sensitive marker of bud neck filament[34], we observed normal Hsl7 localization in *kog1-105* and rapamycin-treated cells (Figure S3A). Actin organization is mainly regulated by rapamycin-insensitive TORC2[1], [35], and actin organization was not affected in *kog1-105* cells (Figure S3B). Localization of Cdc5 was not affected by mutation of *CDC42*, a key player in morphogenesis checkpoint[34]. Cdc28 is required for Cdc5 localization at the bud neck but dispensable for the localization at the SPB[24]. Induction of Clb2 was moderately attenuated in *kog1* cells (Figure 6C), presumably because nuclear localized Cdc5 is involved in *CLB2* expression[26]. However, overexpression of *CLB2* by GAL1 promoter had an only marginal effect on rescue of kog1 mutant (Figure 6E, and Figure S3C). Therefore, we concluded that TORC1-mediated Cdc5 regulation is distinct from the bud neck pathway.

Evidence has been accumulated that dysfunction of Tor results in impairment of overall protein synthesis[1]. We asked whether this decrease of protein synthesis indirectly leads to G2-delay and abnormal localization of Cdc5. First we examined localization of Cdc5 in cells treated with cycloheximide, a potent inhibitor of translation but observed no change in the localization of Cdc5 (Figure S4B). We next examined Cdc5 localization in *cyr1*, *pab1*, and *cdc33* mutants. *CYR1* encodes an adenylate cyclase, playing an important role in protein synthesis[9], [36]. *PAB1* and *CDC33*, encoding Poly(A)-binding protein and eIF-4E, respectively, both perform essential roles in translation initiation[37], [38]. Temperature-sensitive mutants of these essential genes exhibit decreased protein synthesis, which results in early-G1 arrest[9], [37]. In these mutants incubated at the non-permissive temperature GFP-Cdc5 properly localized, such as the SPB and the nucleus (Figure S4B). It is reported that the G1 period of exponentially growing *cdc33-1* cells is twice as long as that of wild-type cells, while the duration of S+G2+M in *cdc33-1* was only slightly (20%) longer than that of wild-type cells[38]. When G1-synchronizing *cdc33-1* cell culture was released at non-permissive temperature (36°C), bud emergence was abolished (Figure S4A)[9] due to prolonged G1. On the other hand, when the *cdc33-1* cells were shifted to 36°C after they exited G1 and formed small buds (105 min), cells completed the entire cell cycle as normal (270–300 min time points). This suggests that impairment of translation initiation does not significantly affect G2 progression. Therefore, we concluded that TORC1 has a distinct role in regulating subcellular localization of Cdc5 and G2/M transition irrespective of its effect on protein synthesis.

**Figure 7:**
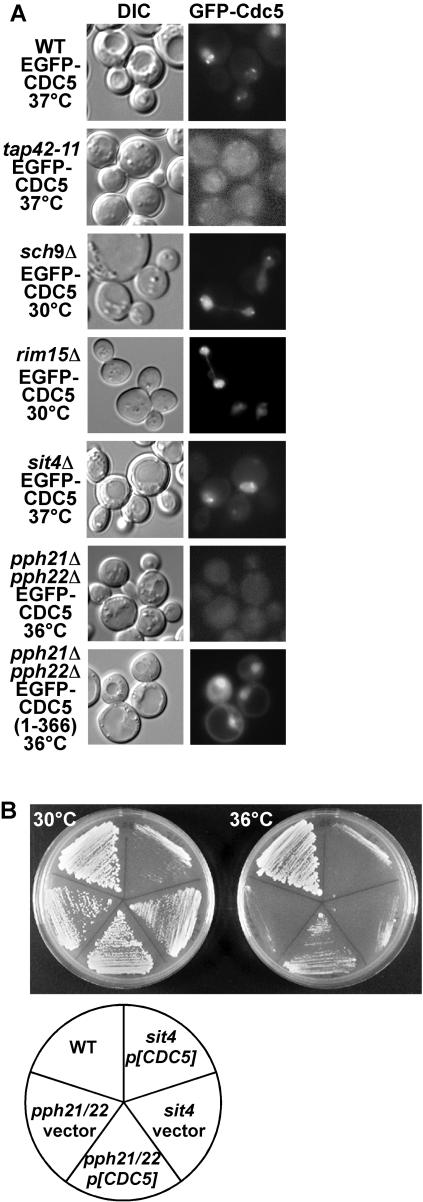
PP2A is involved in localization control of Cdc5. (A) Cells grown in SCD at 30°C (WT (W303-1B), *sch9*Δ (YYK823), *rim15*Δ (YYK831), *sit4*Δ (BY9004), *pph21*Δ*pph22*Δ (BY9943)) or 24°C (*tap42-11*, (CY5755)) expressing EGFP-Cdc5 were shifted to the indicated temperature for 1 h, and EGFP-Cdc5 was observed by fluorescent microscope. (B) Overexpression of CDC5 suppresses growth defect of *pph21*Δ*pph22*Δ. Wild-type, *pph21*Δ*pph22*Δ, and *sit4*Δ cells harboring high-copy CDC5 plasmid were grown on YEPD plates at 30 and 37°C for 2 days. Note that growth defect of *sit4*Δ was exacerbated by CDC5 overexpression.

To determine which factors act downstream of TORC1 to promote nuclear translocation of Cdc5, we examined mutants of genes involved in TORC1 pathway (Figure 7A). Protein kinase A (PKA) is proposed to act downstream of TORC1 for ribosome biogenesis[5]. However, Cyr1, a key component of PKA pathway was not required for Cdc5 localization as described above (Figure S4B). We found that Sch9 was not necessary for proper localization of GFP-Cdc5. Sch9 mediates TORC1-dependent ribosome biogenesis and translation initiation[39], confirming that impaired protein synthesis does not affect Cdc5 localization. Rim15, downstream of PKA and Sch9[39], was also not required for Cdc5 localization. However, mutation of Tap42 prevented the normal localization of Cdc5. Tap42 is involved in the regulation of PP2A and PP2A-related phosphatase Sit4 by TORC1[1], [11]. Abnormal localization of GFP-Cdc5 was observed in a PP2A mutant (*pph21*Δ*pph22*Δ for catalytic subunit), whereas localization appeared normal in a *sit4*Δ strain (Figure 7A). PP2A is known to be required for mitotic entry[40], [41], while Sit4 is involved in G1/S transition[10]. We found that overexpression of *CDC5* rescued the growth defect of *pph21*Δ *pph22*Δ, but not of *sit4*Δ (Figure 7B). GFP-Cdc5(1-366) displayed normal nuclear localization in a PP2A mutant (Figure 7A). These results suggest that the TORC1-PP2A pathway mediates nuclear translocation of Cdc5 via regulation of its intra-molecular interaction.

## Discussion

In this study we present evidence that rapamycin-sensitive TOR (TORC1) pathway plays an important role in G2/M transition. We also demonstrate that TORC1 contributes to subcellular localization of Cdc5. It is widely known that TORC1 is required for G1 progression, and that G1 arrest caused by dysfunction of TOR (e.g. rapamycin treatment) is mainly due to impaired protein synthesis. However, in light of our findings with *pab1* and *cdc33* mutants (Figure S4), another novel TORC1 function than regulating protein synthesis is responsible for G2 progression. Morphogenesis checkpoint is either unlikely to participate in TORC1-mediated control of Cdc5.

PP2A phosphatase has several essential roles in cell growth and proliferation. Loss of PP2A function results in cell cycle arrest at G2[11] and impaired Swe1 degradation[41]. This phenotype concurs with that of *kog1-105* including abnormal localization of Cdc5, suggesting that Tap42-PP2A branch of TORC1 signaling is involved in G2/M transition via regulation of Cdc5.

We have further demonstrated that nucleocytoplasmic translocation of Cdc5 is a prerequisite step for its proper function, e.g. localization and kinase activation. The TORC1-PP2A pathway promotes Cdc5 translocation across the nuclear membrane. Upon TORC1 inactivation, nuclear transport of Cdc5 is inhibited, perhaps because Cdc5 is retained in the cytosol, but this inhibition is overcome by deletion of the C-terminal PBD domain. These results are best explained by assuming that dissociation of the N-terminal catalytic domain (containing the NLS) from the C-terminal PBD is mediated by TORC1 and promotes NLS recognition by the Kap complex.

The C-terminal PBD has a critical role in subcellular localization of Plks. The PBD has a well-studied phosphopeptide-binding activity[42], which helps to recruit Plk to the phosphorylated target proteins at specific cellular locations[23]. Phosphopeptide binding by the PBD disrupts certain intramolecular interactions and lets the kinase domain to be activated[20]. In the resting state, phosphopeptide binding is inhibited by interaction with the kinase domain and the kinase activity is repressed by the association of the PBD[42]. The intra-molecular interaction between the kinase domain and the PBD might thus inhibit nuclear import, recruitment to the target sites, and kinase activity. Our results indicate that nuclear translocation occurs prior to the targeting and the activation, because proper localization and kinase activation of Cdc5 are compromised when its nuclear translocation is prohibited.

Beyond its critical role in G2/M transition, Plk has subsequent essential roles in mitosis[20], [22]. In yeast, Cdc5 is essential for mitotic exit[22]. It is postulated that SPB movement from mother cell to daughter during anaphase sends a signal to Cdc5 at mitotic exit[22]. However, we did not detect any defect at mitotic exit in *kog1-105* or rapamycin-treated cells when they were released from nocodazole arrest (see Figure 2). Therefore, regulation of Cdc5 by TORC1 is restricted at G2/M transition, and it is unlikely that TORC1 regulates Cdc5 at mitosis.

During the course of our study, a report was published suggesting that Tor regulates mitotic entry in the fission yeast, *Schizosaccharomyces pombe* [43]. However, their conclusion is completely opposite to ours; rapamycin treatment stimulates mitotic entry in the fission yeast. We assume that this discrepancy comes from the difference of rapamycin-sensitivity of these two species. The budding yeast used in this study is highly sensitive to rapamycin, whereas cell proliferation of the fission yeast is not inhibited by rapamycin[44], [45]. Presumably rapamycin-FKBP12 complex can bind to and inhibit Tor in the fission yeast cells, because components of Tor complex and FKBP12 are widely conserved among eukaryotes including the two yeasts[46]. We speculate that some downstream pathway(s) of Tor signaling is abrogated in the rapamycin-resistant yeast. Description of rapamycin-resistance in some cancer cell line (e.g. rhabdomyosarcoma Rh30) is reported[47], [48], thus identification of disconnected circuit in TORC1 signaling which confers rapamycin-insensitivity should be quite informative for application of rapamycin as an anti-cancer drug. Judging from the phosphorylation of eIF-2α, Gcn2 kinase is activated by rapamycin in the budding yeast[18], [19] (Figure 1C), though it is not activated in the fission yeast[43] (Nakashima et al. unpublished result). As deletion of *GCN2* or mutation of *SUI2* (encoding eIF2α) confers the budding yeast cell on rapamycin-resistance[18], Gcn2 might be a good candidate for a key factor to determine rapamycin-sensitivity.

Nutrients have two important roles in cell growth. One is to provide material for macromolecule synthesis, and the other is as a signal molecule of Tor pathway. Tor monitors nutrient levels and integrates this information to control cell growth. The fundamental relationship between cell growth and cell cycle was reported by an early landmark study using budding yeast[8]. This study demonstrated that cell growth occurs throughout whole cell cycle, because cell growth is not perturbed in *cdc* mutants (except *cdc25* and *cdc33*) arrested at various stage in cell cycle. Taking their conclusion into consideration, we assume that TOR signaling remains active during whole cell cycle, acting not only to upregulate macromolecular synthesis, but also to transmit nutrient signals to other downstream factors that promote cell cycle progression. Under nutrient-limiting conditions, the best stage to arrest cell growth is at G1. However, after cells exit G1, it is better to slow down cell cycle progression at G2 and to wait for nutrient levels to recover, because mitosis is the most elaborate and energy-consuming event in cell cycle. Our findings suggest that Tor could play another pivotal role in cell cycle monitoring, determining whether cell growth and nutrient conditions are sufficient to permit mitosis.

## Materials and Methods

### Strains, Plasmids, Media and Genetic methods

Yeast strains used in this study are listed in Supporting Information, Table S1. Standard techniques (growth media, cell growth condition, transformation etc.) were used for yeast manipulation[49]. Cloning of 5.4-kb DNA fragment containing *KOG1* gene was carried out by PCR. N-terminal Flag-tagging of *KOG1* and HA-tagging of *TOR1* [2] were done using Quikchange (Stratagene).

### Moleucular Biological Methods

Total RNA was isolated using TRIZOL Reagent (Invitrogen), and northern blot was performed using Random Primer DNA Labeling Kit (TaKaRa) and analyzed by BAS2500 (Fuji Film). Total cell extract was prepared as described below. For immunoblot, anti-HA (16B12, BAbCO), anti-Flag (M2, Sigma), anti-myc (9E10), anti-phospho-eIF2α (pS51, Stressgen), anti-eIF2α (Stressgen), and anti-Cdc2 (PSTAIRE, Santa Cruz) were used.

### Screening of the *kog1* ^ts^ Alleles and the Multicopy Suppressors

Temperature sensitive *KOG1* mutant was conducted by mutagenic PCR. The mutagenized gene was co-transformed into YYK400 cell (*kog1*Δ::*LEU2* pRS316[*KOG1* ^WT^]) with a gapped pRS314[*KOG1*] plasmid. The transformants were replicated on 5-fluoro-orotic acid medium to isolate uracil auxotrophs. One hundred colonies were picked and patched onto SCD-Trp plates, and they were screened for temperature sensitivity at 33, 35 and 37°C. Plasmids recovered from 3 colonies that passed the initial screening were used for the second screening. As a result, we obtained 2 *kog1* temperature-sensitive allele, *kog1-105* and *kog1-117*. We subcloned *KOG1* ^WT^ and *kog1-105* into pRS313 plasmid, transformed them into YYK400, and isolated uracil auxotrophs to generate YYK409 (WT) and YYK410 (*kog1-105*) cells.

Multicopy suppressor screening of *kog1-105* strain was done from 5×10^8^ library transformants as described[35]. Screening was carried out at 36.5°C. As the result of the third screening we obtained 17 multicopy suppressor candidates. Among them, we succeeded in identifying 3 genes, *CDC5*, *ZDS1*, and *RIM15*.

### Cell Synchrony and Fluorescence-activated Cell Sorter (FACS) Analysis

Cell synchronization in G1 was obtained by treatment of exponentially growing *Mat* ***a*** cells with 5 µM α-factor mating pheromone for 2 h. Yeast cells were synchronized in M by treating growing cells with 15 µg/ml nocodazole for 2 h at 30°C. The G1 or M-arrested cells were washed with distilled water three times, released into fresh YEPD or SCD medium, and cells were collected at the indicated time. For FACS analysis, cells were fixed by ethanol for 1 h (or longer), washed by 50 mM sodium citrate in 50 mM Tris-HCl pH 7.5, and suspended in 50 mM Tris-HCl pH 7.5. Cells were treated with 1 mg/ml of RNaseA at 37°C for 1h, then treated with 40 µg/ml of proteinase K at 55°C for 1 h, and washed and suspended in PBS buffer. The resulting cells were stained by 100 µg/ml propidium iodide at room temperature for 1 h. The DNA content of cells was measured on Beckman-Coulter EPICS XL flow cytometer. Nocodazole (Sigma) and rapamycin (Sigma) were used at the final concentration of 15 µg/ml and 200 ng/ml, respectively.

### Immunoprecipitation and Protein Kinase Assay

YEPD-grown cells were collected, washed once with distilled water, and suspended in ice-cold CDC5 lysis buffer (1× PBS, pH 7.4, 1 mM EDTA, 1 mM EGTA, 1 mM Na_3_VO_4_, 15 mM *p*-nitrophenylphosphate (*p*NPP), 1 mM dithiothreitol (DTT), 20 µg/ml leupeptin, 20 µg/ml benzamidine, 10 µg/ml pepstatin A, 40 µg/ml aprotinin, 1 mM PMSF). Cells were broken by vortexing with glass beads for 10 min at 4°C. The beads and cell debris were removed by centrifugation for 5 min at 15,000× g at 4°C, and the supernatant was further clarified by additional 20-min centrifugation. Immunoprecipitation of Cdc5^HA^ was carried out as described[35] using anti-HA ascites. Immunoprecipitated protein was suspended with 27 µl of CDC5 kinase assay buffer (50 mM Mops-KOH pH 7.5, 10 mM MgCl_2_, 2 mM EGTA, 0.4 mM Na_3_VO_4_, 1 mM DTT, 15 mM *p*NPP) containing 6 µg of casein (Sigma). This mixture was preincubated for 3 min at 30°C, the reaction was initiated by adding 3 µl of 0.5 mM [γ-^32^P]ATP (Amersham)(10 µCi/sample). After incubation for 30 min at 30°C, the reaction was terminated by adding 30 µl of 2× SDS-PAGE sample buffer and incubated for 5 min at 65°C. Samples were analyzed by SDS-PAGE and autoradiography as described[35]. *In vitro* protein kinase assay of Cdc28 was performed as described[50].

### Observation of EGFP-tagged Protein

EGFP-tagged Cdc5 protein was expressed by high copy plasmid (pRS424, 425, or 426[EGFP-CDC5]) under control of authentic promoter[23]. EGFP was chromosomally tagged into *TUB1* as described[30]. Cells were grown to early logarithmic phase in synthetic medium (SCD). Cells images were acquired using GFP filter[35]. Actin staining using 0.1 U/µl of rhodamine-phalloidin (Sigma) as described[35]. Nuclear DNA was visualized by 10 µg/ml of DAPI.

## Supporting Information

Figure S1 Rapamycin-sensitive TORC1 pathway is involved in mitotic entry. (A) Percentage of G1 (unbudded cell), S+G2 (budded cell with short spindle), and M phase (large budded cell with long spindle) of experiment shown in Figure 3A and B was shown. (B) Wild-type (WT (JK9-3da)) and rapamycin-resistant *TOR1-1* mutant (JH11-1c) arrested at G1 were released into YEPD. Rapamycin (200 ng/ml) was added at 20 min after release and mating factor was added at 60 min for re-arrest at G1. DNA content was measured by FACS analysis. (C) Wild-type (YYK409) and *kog1-105* (YYK410) cells arrested at G1 were released into YEPD at 37°C. Mating factor was added at 60 min for re-arrest at G1. DNA content was measured by FACS analysis.
(10.00 MB TIF)

Figure S2 Karyopherin-mediated nuclear transport of Cdc5 is essential to Cdc5 function. (A) Karyopherin α and β mediate nuclear localization of Cdc5. Cells expressing GFP-Cdc5 (by pRS426[EGFP-CDC5]) grown in SCD at 24°C (*SRP1* (W303-1B), *srp1-31* (NOY612), and *kap94-14*) or 30°C (*NUP1* (BY4741), *nup1*Δ36 (MRY120)) were observed by fluorescent microscope. As for *kap95-14* mutant, cells grown at 24°C were incubated at 37°C for 1 h. (B) (Left) A conserved NLS domain in Cdc5. We searched classical NLS motifs using PSORTII program and found two putative NLS sequences, ^58^KKKR and ^171^KRRK. The latter site, well conserved among polo-like kinases, was mutated to generate *cdc5*K171A/R172A/R173A (cdc5^3A^). Mutation at the former site did not have any effect on GFP-Cdc5 localization (data not shown). (Right) Cdc5^3A^ protein is expressed at similar level with wild-type Cdc5. HA-tagged Cdc5 protein was detected by immunoblot.
(8.69 MB TIF)

Figure S3 Bud neck organization is not affected by loss of TORC1 function (A) Cells (WT (YYK409) and *kog1-105* (YYK410)) expressing GFP-Hsl7 were incubated at 37°C (*kog1-105*) or with 200 ng/ml rapamycin for 4 h. GFP-Hsl7 localizing at the bud neck was observed by fluorescent microscope. (B) (Top) Cells (WT (YYK409) and *kog1-105* (YYK410)) incubated at 37°C were fixed and actin was stained with rhodamine-phalloidin. (Bottom) EGFP-Cdc5 of *cdc42-1* (YKT366) incubated at 37°C for 2 h was observed by fluorescent microscope. (C) (Top) Wild-type (YYK409), *kog1-105* (YYK410), *kog1-105 swe1*Δ (YYK513), and *kog1-105* cells harboring the indicated plasmids (expressed by their own promoter) were spotted onto YEPD and incubated at the indicated temperature for 2 days. (Bottom) Wild-type (YYK409), *kog1-105* (YYK410) cells harboring the high copy *CDC5* (p[CDC5], expressing Cdc5 by its own promoter) or overexpressing *CLB2* (p[CLB2], expressing under *GAL1* promoter) plasmids were spotted onto YEPD or YEPgalactose (overexpressing Clb2) and incubated at the indicated temperature for 2 days.
(10.07 MB TIF)

Figure S4 Decrease of translation rate is not the primary reason of prolonged G2 or abnormal localization of Cdc5 (A) Temperature-sensitive *cdc33-1* mutant (E17) was arrested at G1 by α-factor at 24°C, and released into YEPD medium. Cell culture was incubated at 24°C (left), 36°C (middle), or growth temperature was shifted from 24°C to 36°C at 105 min (when nearly 50% of the cells exited G1 phase). Budding indices (%) were determined to monitor the cell cycle progression. (B) Exponentially growing cells (WT (YYK409), *cyr1-200* (HM57-2C), *pab1*-F364L (YAS120), and *cdc33-1* (CB101)) expressing EGFP-Cdc5 were incubated as indicated, and EGFP-Cdc5 was observed by fluorescent microscope. CHX, treatment with 10 µg/ml of cycloheximide. As for *cyr1*, *pab1* and *cdc33-1* mutants, about 10% population of the culture at the non-permissive temperature was still budded cells (data not shown).
(9.39 MB TIF)

Table S1 (0.06 MB DOC)
