# Supplementary figures and images for: The Yeast Tor Signaling Pathway Is Involved in G2/M Transition via Polo-Kinase
Source: PLoS One. 2008 May 21;3(5):e2223. doi: 10.1371/journal.pone.0002223 (PMC2375053; doi:10.1371/journal.pone.0002223)

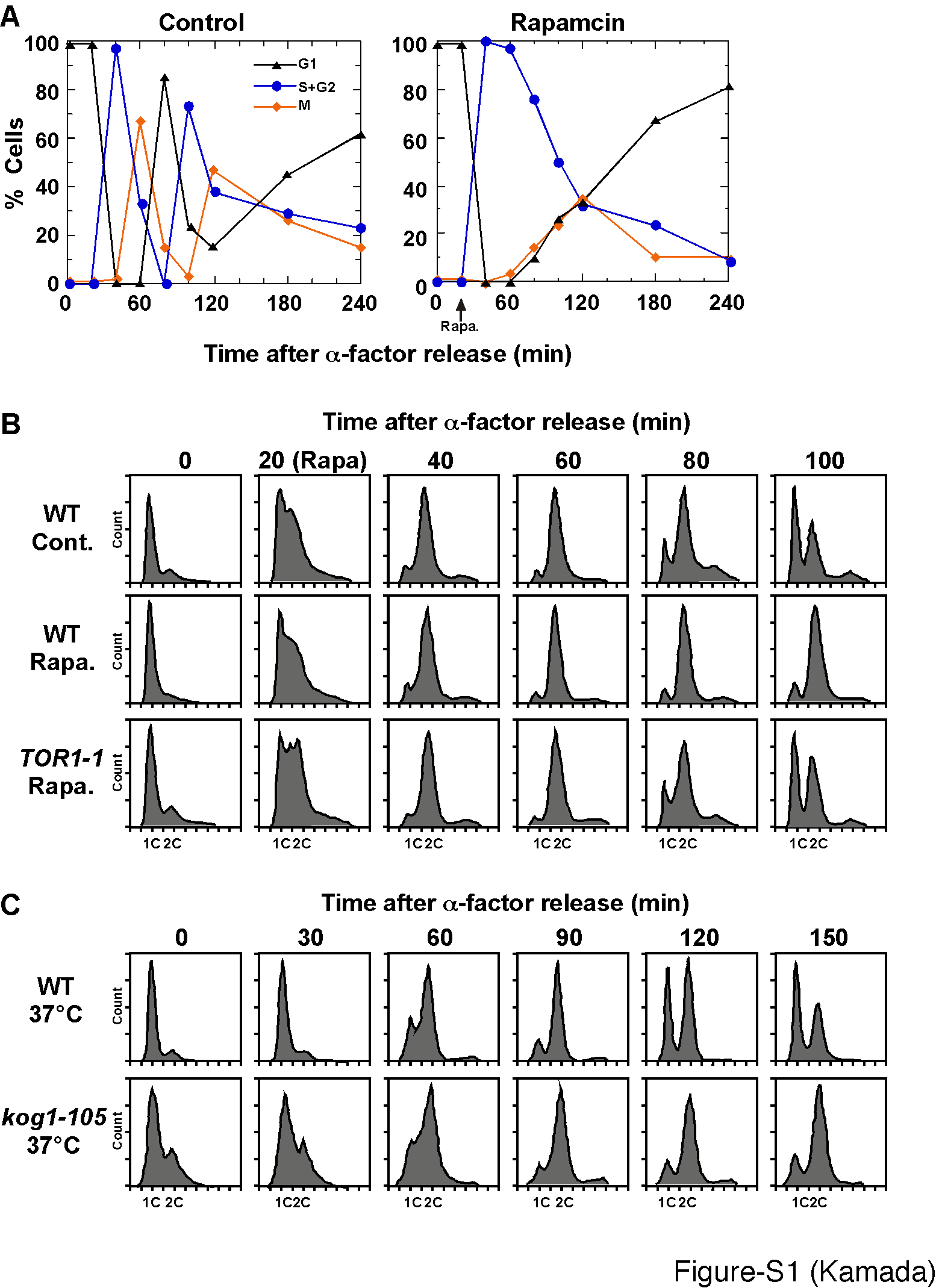

Supplement: Figure S1 — Rapamycin-sensitive TORC1 pathway is involved in mitotic entry. (A) Percentage of G1 (unbudded cell), S+G2 (budded cell with short spindle), and M phase (large budded cell with long spindle) of experiment shown in Figure 3A and B was shown. (B) Wild-type (WT (JK9-3da)) and rapamycin-resistant TOR1-1 mutant (JH11-1c) arrested at G1 were released into YEPD. Rapamycin (200 ng/ml) was added at 20 min after release and mating factor was added at 60 min for re-arrest at G1. DNA content was measured by FACS analysis. (C) Wild-type (YYK409) and kog1-105 (YYK410) cells arrested at G1 were released into YEPD at 37°C. Mating factor was added at 60 min for re-arrest at G1. DNA content was measured by FACS analysis. (10.00 MB TIF) [file pone.0002223.s001.tif]

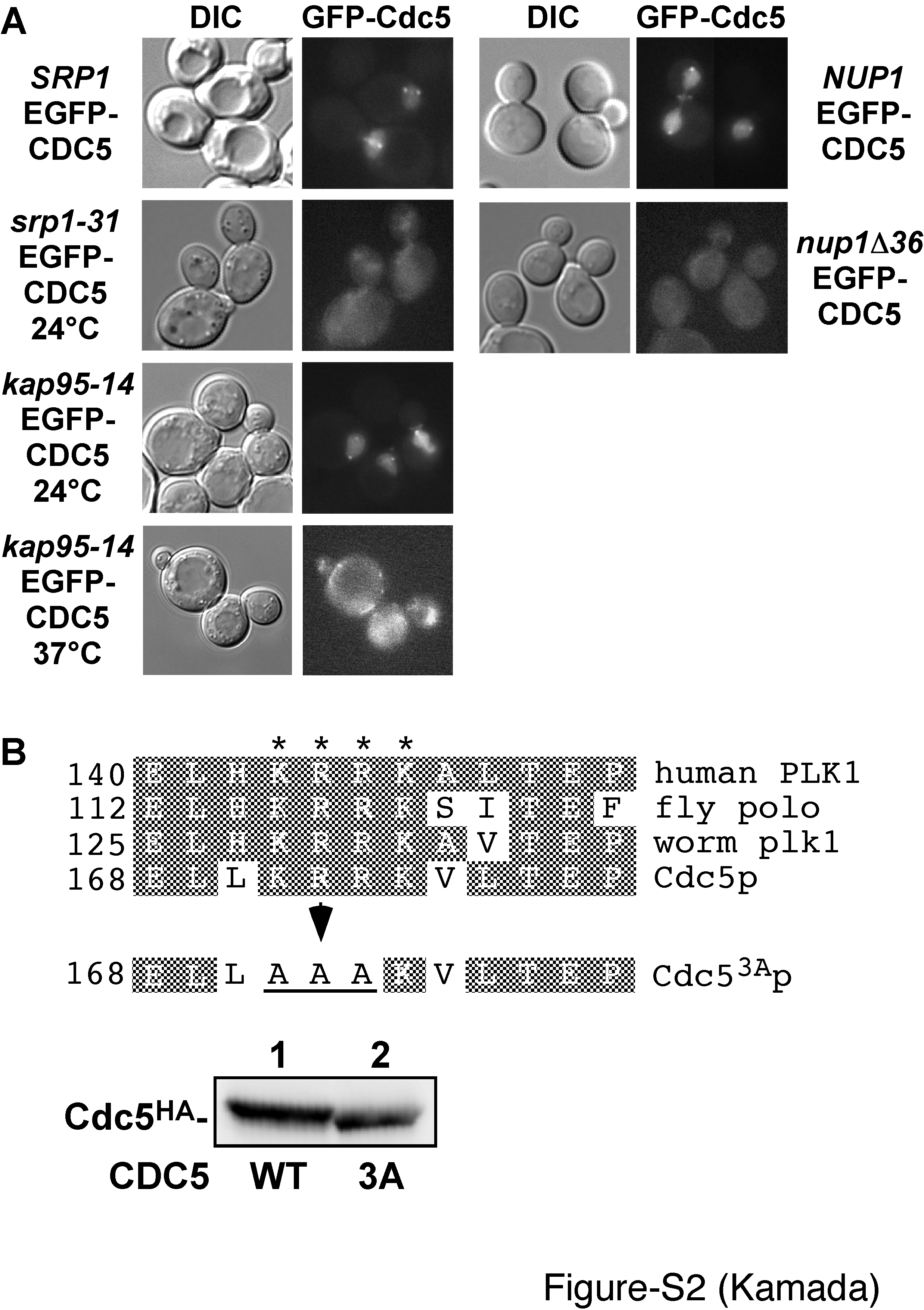

Supplement: Figure S2 — Karyopherin-mediated nuclear transport of Cdc5 is essential to Cdc5 function. (A) Karyopherin α and β mediate nuclear localization of Cdc5. Cells expressing GFP-Cdc5 (by pRS426[EGFP-CDC5]) grown in SCD at 24°C (SRP1 (W303-1B), srp1-31 (NOY612), and kap94-14) or 30°C (NUP1 (BY4741), nup1Δ36 (MRY120)) were observed by fluorescent microscope. As for kap95-14 mutant, cells grown at 24°C were incubated at 37°C for 1 h. (B) (Left) A conserved NLS domain in Cdc5. We searched classical NLS motifs using PSORTII program and found two putative NLS sequences, 58KKKR and 171KRRK. The latter site, well conserved among polo-like kinases, was mutated to generate cdc5K171A/R172A/R173A (cdc53A). Mutation at the former site did not have any effect on GFP-Cdc5 localization (data not shown). (Right) Cdc53A protein is expressed at similar level with wild-type Cdc5. HA-tagged Cdc5 protein was detected by immunoblot. (8.69 MB TIF) [file pone.0002223.s002.tif]

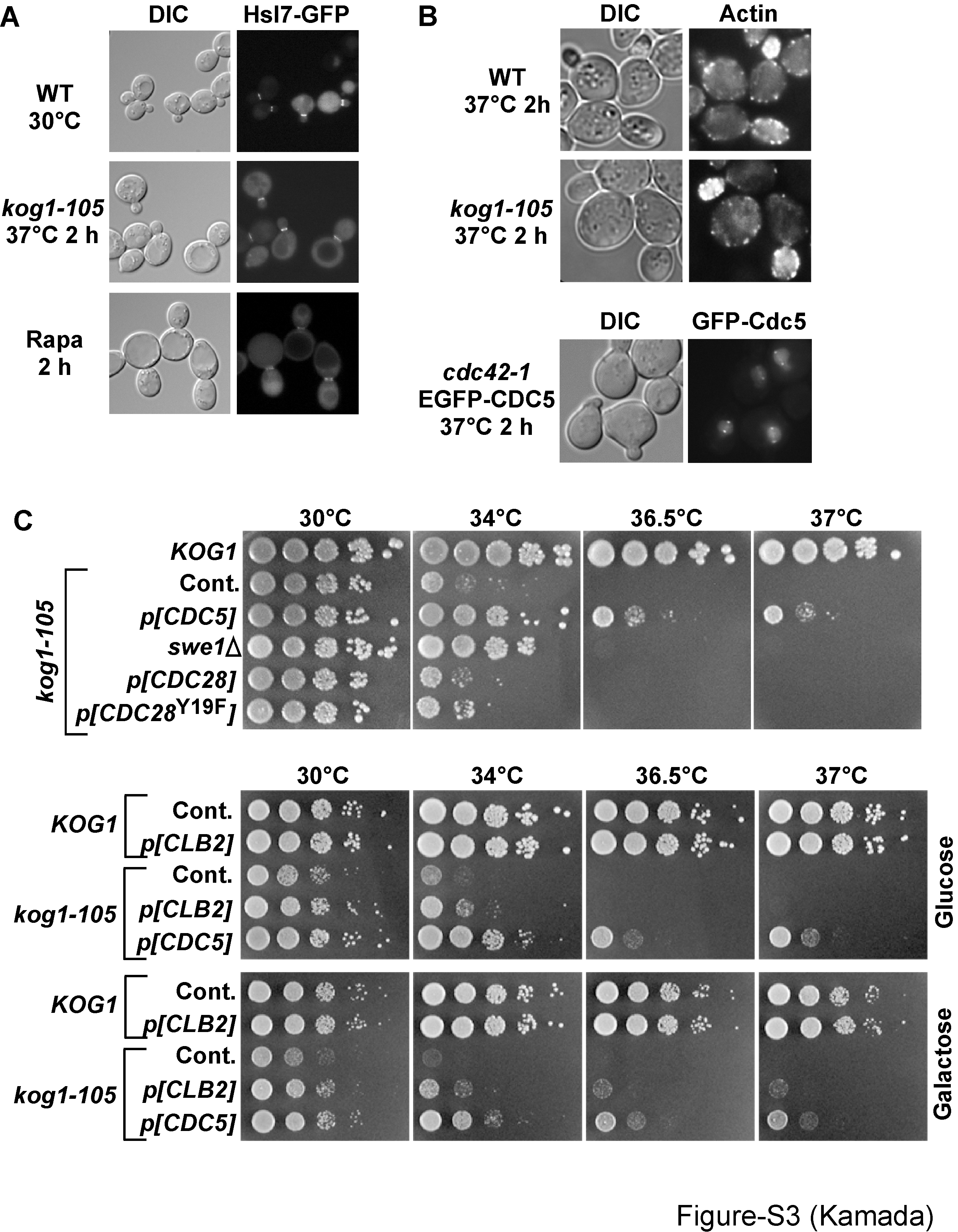

Supplement: Figure S3 — Bud neck organization is not affected by loss of TORC1 function (A) Cells (WT (YYK409) and kog1-105 (YYK410)) expressing GFP-Hsl7 were incubated at 37°C (kog1-105) or with 200 ng/ml rapamycin for 4 h. GFP-Hsl7 localizing at the bud neck was observed by fluorescent microscope. (B) (Top) Cells (WT (YYK409) and kog1-105 (YYK410)) incubated at 37°C were fixed and actin was stained with rhodamine-phalloidin. (Bottom) EGFP-Cdc5 of cdc42-1 (YKT366) incubated at 37°C for 2 h was observed by fluorescent microscope. (C) (Top) Wild-type (YYK409), kog1-105 (YYK410), kog1-105 swe1Δ (YYK513), and kog1-105 cells harboring the indicated plasmids (expressed by their own promoter) were spotted onto YEPD and incubated at the indicated temperature for 2 days. (Bottom) Wild-type (YYK409), kog1-105 (YYK410) cells harboring the high copy CDC5 (p[CDC5], expressing Cdc5 by its own promoter) or overexpressing CLB2 (p[CLB2], expressing under GAL1 promoter) plasmids were spotted onto YEPD or YEPgalactose (overexpressing Clb2) and incubated at the indicated temperature for 2 days. (10.07 MB TIF) [file pone.0002223.s003.tif]

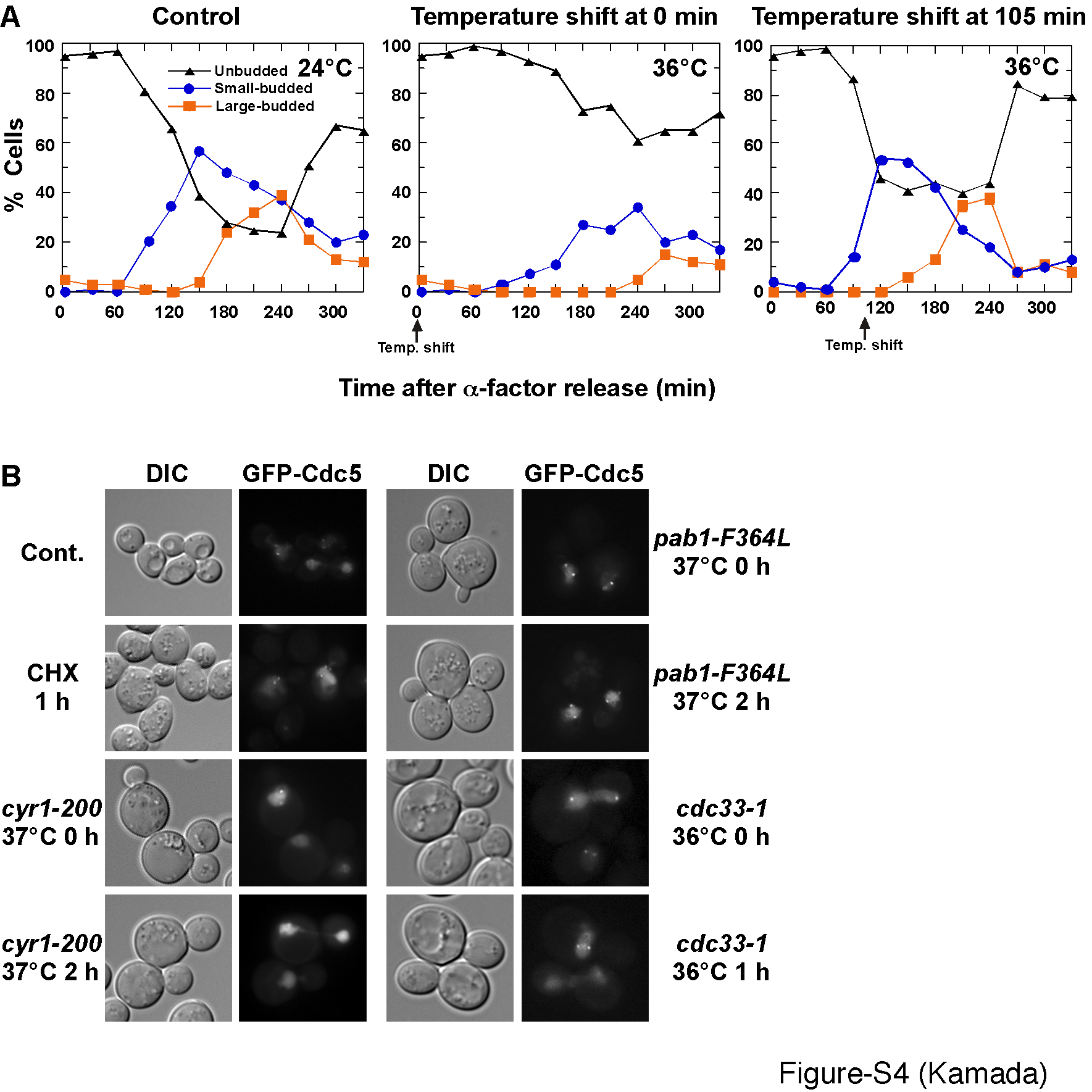

Supplement: Figure S4 — Decrease of translation rate is not the primary reason of prolonged G2 or abnormal localization of Cdc5 (A) Temperature-sensitive cdc33-1 mutant (E17) was arrested at G1 by α-factor at 24°C, and released into YEPD medium. Cell culture was incubated at 24°C (left), 36°C (middle), or growth temperature was shifted from 24°C to 36°C at 105 min (when nearly 50% of the cells exited G1 phase). Budding indices (%) were determined to monitor the cell cycle progression. (B) Exponentially growing cells (WT (YYK409), cyr1-200 (HM57-2C), pab1-F364L (YAS120), and cdc33-1 (CB101)) expressing EGFP-Cdc5 were incubated as indicated, and EGFP-Cdc5 was observed by fluorescent microscope. CHX, treatment with 10 µg/ml of cycloheximide. As for cyr1, pab1 and cdc33-1 mutants, about 10% population of the culture at the non-permissive temperature was still budded cells (data not shown). (9.39 MB TIF) [file pone.0002223.s004.tif]
