# Supplementary material for: The Yeast Tor Signaling Pathway Is Involved in G2/M Transition via Polo-Kinase
Source: PLoS One. 2008 May 21;3(5):e2223. doi: 10.1371/journal.pone.0002223 (PMC2375053; doi:10.1371/journal.pone.0002223)
Supplement: Table S1 — (0.06 MB DOC) [file pone.0002223.s005.doc]

Table S1 Yeast strains used in this study

| Strain | Genotype | Source |
| --- | --- | --- |
| W303-1B | *Mat****a*** *ade2 his3 leu2 trp1 ura3 can1* | Laboratory stock |
| W303-1A | *Mat****α*** *ade2 his3 leu2 trp1 ura3 can1* | Laboratory stock |
| BY4741 | *Mat****a*** *his3 leu2 met15 ura3* | Laboratory stock |
| JK9-3da | *Mat****a*** *his4 leu2-3,112 trp1 ura3 rme1* | M. N. Hall |
| JH11-1c | JK9-3da *TOR1-1* | M. N. Hall |
| SH121 | JK9-3da *tor2::ADE2-3* YCplac111[*tor2-21*] | M. N. Hall |
| SH221 | JK9-3da *tor1::HIS3-3 tor2::ADE2-3*  YCplac111[*tor2-21*] | M. N. Hall |
| YYK400 | W303-1B *kog1Δ::LEU2* pRS316[*KOG1*] | This study |
| YYK409 | W303-1B *kog1Δ::LEU2* pRS313[*KOG1*] | This study |
| YYK410 | W303-1B *kog1Δ::LEU2* pRS313[*kog1-105*] | This study |
| YYK513 | YYK410 *swe1Δ::Hyg^R^* | This study |
| YYK540 | YYK409 *swe1Δ::HIS3::SWE1-9myc::URA3* | This study |
| YYK541 | YYK410 *swe1Δ::HIS3::SWE1-9myc::URA3* | This study |
| YYK834 | W303-1A *kog1Δ::LEU2* pRS313[*kog1-105*]  *TUB1*-GFP::*LEU2* | This study |
| YYK861 | YYK409 *CLB2::CLB2-HA* | This study |
| YYK862 | YYK410 *CLB2::CLB2-HA* | This study |
| YYK831 | BY4741 *rim15*Δ::KanMX | Laboratory stock |
| YYK823 | BY4741 *sch9*Δ::KanMX | This study |
| YAN86 | W303-1B *kog1Δ::LEU2* pRS314[^Flag^*KOG1*] | This study |
| YAN103 | W303-1B *kog1Δ::LEU2* pRS314[^Flag^*kog1-105*] | This study |
| KLY1548 | W303-1B *ADE3* | Laboratory stock |
| KLY2156 | KLY1548 *cdc5-1* | Laboratory stock |
| KLY4206 | KLY1548 *TUB1*-GFP::*LEU2* | Laboratory stock |
| NOY612 | W303-1B *srp1-31* | M. Nomura |
| CY5755 | W303-1A *tap42Δ::TRP1* pCEN-LEU2[*tap42-11*] | K. Arndt |
| BY9004 | W303-1B *sit4Δ::CgHIS3* | National-Bio-Resource-Project |
| BY9943 | W303-1B *pph21Δ::CgTRP1 pph22Δ::CgHIS3* | National-Bio-Resource-Project |
| Kap95-14 | *Mat****a*** *his3-200 leu2-3112 lys2-801 trp1-1*  *ura3-52 kap95::HIS3* pTRP1[*kap95-14*] | J. D. Aitchison |
| MRY120 | BY4741 *nup1Δ36* | M. Rexach |
| YAS120 | *Mat****α*** *ade2 his3 leu2 trp1 ura3 pab1::HIS3*  pCEN-TRP1[*pab1*-F364L] | A. Sachs |
| E-17 | *Mat****a*** *ade1 ade2 gal1 his7 lys2 tyr1 ura1 cdc33-1* | H. Iida |
| CB101 | *Mat****α*** *leu1 trp1 ura3 cdc33-1* | K. Matsumoto |
| HM-57-2C | *Mat****a*** *leu2 met4 trp1 ura3 ras1::HIS3 cyr1-230* | H. Mitsuzawa |
| YKT366 | *Mat****α*** *gal2 his4 leu1 trp1 ura3 cdc42-1* | K. Tanaka |
